# Major Organic Involvement in Women with Fabry Disease in Argentina

**DOI:** 10.1155/2018/6515613

**Published:** 2018-05-21

**Authors:** Fernando Perretta, Norberto Antongiovanni, Sebastián Jaurretche

**Affiliations:** ^1^Servicio de Terapia Intensiva del Hospital Dr. Enrique Erill de Escobar, Buenos Aires, Argentina; ^2^GINEF (Grupo de Investigación Nefrológica en la Enfermedad de Fabry), Buenos Aires, Argentina; ^3^Centro de Infusión y Estudio de Enfermedades Lisosomales del Instituto de Nefrología Clínica Pergamino, Buenos Aires, Argentina; ^4^Centro de Neurociencias Los Manantiales, Grupo Gamma Rosario, Santa Fe, Argentina; ^5^Cátedra de Biofísica y Fisiología, Instituto Universitario Italiano de Rosario, Santa Fe, Argentina

## Abstract

Fabry disease (FD) is an X-linked lysosomal storage disorder resulting from the deficiency or absence of the alpha galactosidase A enzyme. Organic involvement in men is well known, but in women it is controversial, partly due to the random X-chromosomes inactivation (Lyon hypothesis). The aim of this study was to describe the organic involvement in women at the time of FD diagnosis. A descriptive, cross-sectional and multicenter study was carried out. Thirty-five women with FD from three reference centers in Argentina were evaluated. The mean age of the whole group (*n* = 35) was 26.6 ± 16.9 years; 22 were adult (over 18) and 13 were paediatric patients. Enzymatic activity was performed in 29/35 patients, which was normal in 24/29 (82.8%). Seven different mutations of the GLA gene were found. The results showed urinary protein loss (45.7%) and decreased glomerular filtration rate (31.4%), mainly in adults. And also, cornea verticillata (56.5%), peripheral neuropathy (51.4%), cardiovascular manifestations (31.4%), hearing loss (20%), angiokeratomas (20%), central nervous system (17.1%), and gastrointestinal involvement (14.3%). Organic compromise in females with FD may be as severe as in men. This analysis has demonstrated a significant proportion of women with signs, symptoms, and major organic involvement at FD diagnosis.

## 1. Introduction

Fabry disease (FD, OMIM #301500) is caused by the lysosomal accumulation of complex glycosphingolipids, mainly globotriaosylceramide (Gb3) and its metabolites [1]. This deposit triggers physiopathogenic pathways in the vascular endothelium and cells of different tissues (cardiac, renal, and nervous among others) that lead to cell death, with progression to fibrosis and irreversible organic damage [2, 3]. The storage of Gb3 is due to the deficient or null activity of *α*-galactosidase A enzyme (*α*-galA, EC 3.2.1.22). The GLA gene, which encodes *α*-galA, is located on the X-chromosome (Xq22.1), whereby practically all men carrying a genetic mutation (hemizygous) develop the disease, while women (heterozygotes) exhibit a wide variability in the severity of their phenotype, mainly due to the random X-chromosomes inactivation in each of their cells (Lyon hypothesis) [4]. The symptoms intensity will depend mostly on the residual activity of the *α*-galA enzyme. The dosage of *α*-galA in dried blood spot on filter paper is useful in males and a decrease activity confirms the disease, while in females the molecular study is required because of the possibility of false negatives.

FD manifestations are multisystemic and begin in childhood, reaching severe impairment in the third or fourth decade of life. The main signs and symptoms of the disease are acroparesthesias in hands and feet, gastrointestinal disorders, angiokeratomas, dyshidrosis, intolerance to exercise and heat, hearing loss, arrhythmias, hypertrophic cardiomyopathy, cerebrovascular accidents, and renal failure [5, 6]. It has been described that male patients had a higher frequency of symptoms at an earlier age than females [7].

Although FD has been a known pathology for more than 100 years, over the last decade the prognosis has changed significantly due to the possibility of enzyme replacement therapy (ERT). Multiple studies have demonstrated that ERT effectively reduces Gb3 accumulation, improves anhidrosis, peripheral nerve function, gastrointestinal symptoms, and acroparesthesias, and can stabilize kidney function [8–12].

In 2001, it was described that most women were asymptomatic and had a completely normal quality of life or developed only mild manifestations of the disease [13]. However, several studies have reported that heterozygous women may develop severe symptoms with risk of premature death [14, 15].

FD is panethnic and, given its low incidence, there is no accurate information regarding its prevalence, ranging from 1:40,000 men to 1:117,000 live births [13, 16]. Due to the great phenotypic and symptoms variability, it is difficult to perform a precise diagnosis. Like other rare diseases, it has been difficult to understand the natural progression of FD because of the scarcity of clinical information, especially in women.

The aim of this study was to describe the organic involvement in women at the time of FD diagnosis. In addition, organic involvement according to age was evaluated.

## 2. Material and Methods

A descriptive, cross-sectional, and multicenter study was carried out. Thirty-five women with FD from 3 reference centers in Argentina were evaluated: Critical Care Unit, Hospital Dr. Enrique Erill, Escobar City, State of Buenos Aires; Neurosciences Center Los Manantiales, Gamma Group, Rosario City, State of Santa Fe; and Infusion Center of Lysosomal Diseases, Nephrology Institute of Pergamino, State of Buenos Aires. Plasma *α*-gal-A activity was performed on filter paper by fluorometric method in Dr. N. A. Chamoles/FESEN Laboratory (Buenos Aires, Argentina) [17] and the mutational study by MLPA (multiplex ligation-dependent probe amplification) and sequencing in Medical Genetics Laboratories-Baylor College of Medicine (Houston, TX, USA) [18, 19].

Renal involvement was determined by the loss of proteins in urine (albuminuria/proteinuria) and/or the decrease in the estimated glomerular filtration rate (eGFR). The eGFR was calculated using serum creatinine by CKD-EPI formula in adult patients and Schwartz formula in pediatric patients [20, 21]. Both formulas have been recently validated in patients with FD [22, 23]. Values greater than 125 ml/min/1.73 m^2^ were defined as glomerular hyperfiltration [22]. The GFR categories were defined according to KDIGO 2012 guidelines [24]. Pathological albuminuria was considered at values > 30 mg/day or >30 mg/g creatinine and proteinuria at values > 300 mg/day or >300 mg/g creatinine, in at least 2 different urine samples in all cases.

Central nervous system (CNS) involvement was determined by the prevalence of transient ischemic attacks (TIAs), hemorrhagic or ischemic strokes, or the presence of silent white matter lesions on nuclear magnetic resonance. The peripheral neuropathy was defined by the presence of discomfort in hands and feet, with paroxysmal burning pains of the palms and soles.

Cardiovascular compromise was determined by left ventricular hypertrophy (LVH) and/or arrhythmia. LVH was evaluated by transthoracic echocardiogram and arrhythmia by 12-lead electrocardiogram (ECG). Thickness of the interventricular septum (IVS) and left ventricle posterior wall (LVPW) were considered to be normal between 6 and 11 mm [25].

The cornea verticillata was evaluated by ophthalmologic examination including slit lamp and the presence of angiokeratomas by dermatologists with experience in FD. Gastrointestinal involvement centered on subjective complaints of abdominal pain and bowel dysmotility, not explained by other causes than FD. Hearing loss was determined with tonal audiometry by an otorhinolaryngologist.

The study was conducted in compliance with the Declaration of Helsinki and was approved by each local Ethics Committee. Written informed consent was obtained from patients.

### 2.1. Statistical Analyses

Categorical variables were expressed as number (percentages) and continuous variables as mean ± standard deviation (SD). The contingency tables were analyzed with Fisher exact or Chi^2^ test. Differences were considered significant if *p* < 0.05. Statistical analyses were performed with GraphPad Prism 2.0 (GraphPad, San Diego, USA).

## 3. Results

### 3.1. Main Characteristics

The mean age of the whole group (*n* = 35) was 26.6 ± 16.9 years; 22 were adult women (>18 years) with mean age of 37.0 ± 12.2 years and 13 were pediatric patients (<18 years) with mean age of 9.2 ± 4.3 years. Two index cases were detected; both are adult women aged 28 and 55. Seven different mutations of the GLA gene were found (Table 1). The most frequent was E398X in 14 patients (40.0%): 6 pediatric patients and 8 adults. It was followed by p.L415P, which was found in 11 patients (31.4%): 7 pediatric patients and 4 adults. No other mutation was found in pediatric patients. All of them are classical mutations, except for p.R301Q (*n* = 1), which is described as classic phenotype and also as late onset variant of FD (http://fabry-database.org/mutants).

Plasma *α*-gal-A levels were performed in 29/35 patients (82.9%) and was normal in 24/29 (82.8%). The results of 6 patients are not available. The decreased measures corresponded to the mutations E398X (*n* = 2) and p.L415P (*n* = 3).

### 3.2. Renal Involvement

Tables 2 and 3 describe the eGFR (ml/min/1.73 m^2^) and urinary protein loss (albuminuria/proteinuria) of the study population. Nine patients (25.7%) had normal renal function at the time of FD diagnosis, but 15 patients (42.9%) presented glomerular hyperfiltration. Decreased eGFR (≤90 ml/min/1.73 m2) was found in 11 patients (31.4%), mainly in adults (10 adults and 1 pediatric patient; Fisher's test, *p* = 0.0284). Of the adult patients 9 were of category G2 and 1 of category G3a, while the pediatric patient was in category G2 according to KDIGO 2012 guidelines.

Pathologic urinary protein loss (albuminuria/proteinuria) was observed in 16/35 (45.7%) with predominance of adult patients (Fisher's test, *p* = 0.0125). Albuminuria was detected in 12 adults and in 1 pediatric patient (37.1%) and proteinuria in 2 adults and one child (8.6%).

### 3.3. Other Organic Involvement

Table 4 shows different organic involvement. One of the most frequent compromises was the peripheral neuropathy in 18 patients (51.4%) with predominance of adults (15/18, Fisher's test, *p* = 0.0153). The CNS was affected in 17.1% of patients without difference according to the age. The only pediatric patient affected had silent white matter lesions on nuclear magnetic resonance. Cardiovascular manifestations (left ventricular hypertrophy and/or arrhythmia) were observed in 11 patients (31.4%), and statistical difference according to age was observed only in left ventricular hypertrophy (Fisher's test, *p* = 0.0312). We detected 1 adult patient with ventricular arrhythmia, another with atrial fibrillation, and 2 pediatric patients with sinus bradycardia. Hearing loss was found in 20% of FD women, all adult patients (Fisher's test, *p* = 0.0312). The other organic involvement (cornea verticillata 56.5%, angiokeratomas 20% and gastrointestinal involvement 14.3%) did not show differences according to age. However, cornea verticillata and angiokeratomas were the most frequent signs in pediatric patients.

## 4. Discussion

In FD, like other X-linked genetic disorders, complications are less frequent and more variable in severity in women than in men, although some women may present similar phenotypes as males [6, 15, 26].

Enzyme activity in women with FD is usually in the lower normal range owing to the X-chromosomal inheritance pattern of the disease [27]. Therefore, the determination of plasmatic *α*-gal-A activity in women is often inconclusive, so the molecular study is recommended. The X-chromosome inactivation (Lyon hypothesis) could explain the high percentage of normal enzymatic dosages found in our study population. This chromosomal inactivation significantly impacts the phenotype and natural history of FD in females [28].

Nephropathy is one of the major complications of FD [29]. Fabry nephropathy is characterized by variable levels of disease severity, with an overall rate of progression of Chronic Kidney Disease (CKD) very similar to diabetic nephropathy, with evidences suggesting that untreated patients usually develop End Stage Renal Disease (ESRD) into their 50s [30–32]. Usually, untreated affected males show three clinical phases of Fabry nephropathy [2]. The first occurs in childhood and adolescence, and it is distinguished by glomerular hyperfiltration. The second clinical phase is characterized by renal involvement with proteinuria, lipiduria, Malta crosses cristals in the urine sediment examined by polarized microscopy, impairment in the urine concentrating or diluting ability, and other renal dysfunctions. The third phase presents severe renal disease and involvement of vascular, cardiac, and cerebral systems. It is known that renal involvement is more frequent in men than in women. In our report we highlight a high percentage of women with renal hyperfiltration, predominantly in pediatric patients, as probable glomerular compensation for Fabry nephropathy. On the other hand, the decreased eGFR was detected in one-third of our population, with statistical significance in adult patients over pediatric female patients, without specific therapy for FD. This could be interpreted as the natural progression of FD nephropathy. A limitation of this study is that the GFR was assessed by formula and not measured.

Urinary protein excretion is strongly associated with renal disease progression in men and women with FD [33]. Albuminuria and proteinuria are common elements of a progressive Fabry nephropathy that may reach nephrotic range [34]. In this study, almost half of the female patients showed urinary protein loss (albuminuria/proteinuria), mainly in the adult population, at the time of FD diagnosis. Albuminuria was the most frequent nephropathy sign detected.

On the other hand, we found that women frequently suffer from neuropathic pain, cornea verticillata, hearing loss, angiokeratomas, and cardiovascular and CNS involvement. Gastrointestinal manifestations were not frequent (14.3%). However, severe abdominal cramping was reported in 39% (16/41) and diarrhea was reported in 43% (18/42) [15].

In pediatric patients we observed mainly cornea verticillata and angiokeratomas. In addition, neuropathic pain and arrhythmias were found as early clinical manifestations. These signs and symptoms are often not recognized as FD, delaying diagnosis and treatment.

One-third of our patients showed cardiovascular involvement determined by LVH and/or arrhythmia. Wilcox et al. [26] reported that cardiac compromise was the most severe manifestation among women in their cohort. Cardiac involvement, with LVH and structural valve abnormalities, is very common and worsens with age in females who are heterozygous for FD [35]. In our study group we found similar number of patients with arrhythmias according to age, but left ventricular hypertrophy was only found in adult FD women. Hybrid ^18^F-fluorodeoxyglucose (FDG) positron emission tomography and magnetic resonance imaging may differentiate mature fibrosis or scar from fibrosis associated to active inflammation in patients with FD, even in nonhypertrophic stage. In FD female patients, focal ^18^F-FDG uptake represents an early sign of disease-related myocardial damage and is associated with impaired left ventricular longitudinal function [36].

Neurological manifestations (CNS and peripheral neuropathy) were observed in two-thirds of the study population. Similar data was previously reported by Deegan et al. [37]. The peripheral neuropathy was more frequent than the CNS involvement. As often seen in affected males, recurrent burning sensations in the extremities (acroparesthesias) were also one of the first symptoms in FD women [38]. Although other authors have reported that neuropathic pain is the most frequent symptom at a younger age [7, 39], we found that acroparesthesias were predominant in adult FD female patients.

Treatment options for patients with FD include long-term ERT in addition to supportive management. ERT using recombinant human *α*-galactosidase A is intended to reduce disease severity and delay the progression of the disorder. ERT is available in the form of agalsidase alfa (Replagal®, Shire HGT, Inc., Cambridge, MA, USA) and agalsidase beta (Fabrazyme®, Sanofi Genzyme, Cambridge, MA, USA). Agalsidase alfa is given at 0.2 mg/kg body weight every other week by intravenous infusion and is approved in many countries throughout the world, though not by the US Food and Drug Administration. Agalsidase beta is administered at 1.0 mg/kg body weight once every 2 weeks as an intravenous infusion and is approved in Europe, the USA, and many other countries. Recently, the oral small-molecule pharmacological chaperone migalastat (Galafold™; Amicus Therapeutics, Cranbury, NJ, USA) received approval in Europe and Canada for the treatment of a subset of Fabry patients with mutations predicted as “amenable” according to their response to migalastat in an in vitro assay [40].

In conclusion, women with FD suffer from significant multisystemic disease which can lead to deterioration in the quality of life and risk of premature death. This analysis has demonstrated a significant proportion of women with neuropathic pain, hearing loss, angiokeratomas, cornea verticillata, and major organic involvement such as heart, CNS, and kidney, at FD diagnosis. Females should be regularly monitored to determine the timing of enzyme replacement therapy initiation, which must be early without waiting for advanced organic compromise.

## Figures and Tables

**Table 1:** GLA gene mutations in women with FD (*n* = 35).

Mutation	All (*n*, %)	Adults	Pediatrics
E398X	14 (40.0)	8	6
p.L415P	11 (31.4)	4	7
A292T	4 (11.4)	4	-
c.680G>A	3 (8.5)	3	-
del 3&4 exons	1 (2.9)	1	-
p.W81X	1 (2.9)	1	-
p.R301Q	1 (2.9)	1	-

Total	35 (100)	22	13

**Table 2:** eGFR ml/min/1.73 m^2^.

	All (*n*, %)	Adults	Pediatrics
Normal	9 (25.7%)	6	3
Hyperfiltration	15 (42.9%)	6	9
≤90	11 (31.4%)	10	1

Total	35 (100%)	22	13

**Table 3:** Loss of protein in urine.

	All (*n*, %)	Adults	Pediatrics
Normal	19 (54.3%)	8	11
Albuminuria	13 (37.1%)	12	1
Proteinuria	3 (8.6%)	2	1

Total	35 (100%)	22	13

**Table 4:** Other organic involvements.

	All (*n*, %)	Adults	Pediatrics	*p*
Central nervous system	6/35 (17.1%)	5	1	ns
Peripheral neuropathy	18/35 (51.4%)	15	3	0.0153
Left ventricular hypertrophy	7/35 (20.0%)	7	0	0.0312
Arrhythmia	4/35 (11.4%)	2	2	ns
Cornea verticillata^#^	13/23 (56.5%)	8	5	ns
Gastrointestinal involvement	5/35 (14.3%)	4	1	ns
Angiokeratomas	7/35 (20%)	3	4	ns
Hearing loss	7/35 (20%)	7	0	0.0312

^#^It was evaluated in 23 FD women.

## Data Availability

The data used to support the findings of this study are available from the corresponding author upon request.
